# Lycopene as a Therapeutic Agent against Aflatoxin B1-Related Toxicity: Mechanistic Insights and Future Directions

**DOI:** 10.3390/antiox13040452

**Published:** 2024-04-11

**Authors:** Meng Li, Shusheng Tang, Xinyan Peng, Gaurav Sharma, Shutao Yin, Zhihui Hao, Jichang Li, Jianzhong Shen, Chongshan Dai

**Affiliations:** 1 National Key Laboratory of Veterinary Public Health and Safety, College of Veterinary Medicine, China Agricultural University, Beijing 100193, China; s20193050723@cau.edu.cn (M.L.); tssfj@cau.edu.cn (S.T.); yinshutao@cau.edu.cn (S.Y.); haozhihui@cau.edu.cn (Z.H.); 2 College of Life Sciences, Yantai University, Yantai 264000, China; pengxinyan2006@ytu.edu.cn; 3 Cardiovascular and Thoracic Surgery, Advanced Imaging Research Center, University of Texas Southwestern Medical Center, Dallas, TX 75390, USA; gaurav.sharma@utsouthwestern.edu; 4 College of Veterinary Medicine, Northeast Agricultural University, 600 Changjiang Road, Xiangfang District, Harbin 150030, China; lijichang@neau.edu.cn

**Keywords:** aflatoxin B1 (AFB1), lycopene, molecular mechanism, safety, clinical prospects

## Abstract

Aflatoxin (AFT) contamination poses a significant global public health and safety concern, prompting widespread apprehension. Of the various AFTs, aflatoxin B1 (AFB1) stands out for its pronounced toxicity and its association with a spectrum of chronic ailments, including cardiovascular disease, neurodegenerative disorders, and cancer. Lycopene, a lipid-soluble natural carotenoid, has emerged as a potential mitigator of the deleterious effects induced by AFB1 exposure, spanning cardiac injury, hepatotoxicity, nephrotoxicity, intestinal damage, and reproductive impairment. This protective mechanism operates by reducing oxidative stress, inflammation, and lipid peroxidation, and activating the mitochondrial apoptotic pathway, facilitating the activation of mitochondrial biogenesis, the endogenous antioxidant system, and the nuclear factor erythroid 2-related factor 2 (Nrf2)/kelch-like ECH-associated protein 1 (KEAP1) and peroxisome proliferator-activated receptor-γ coactivator-1 (PGC-1) pathways, as well as regulating the activities of cytochrome P450 (CYP450) enzymes. This review provides an overview of the protective effects of lycopene against AFB1 exposure-induced toxicity and the underlying molecular mechanisms. Furthermore, it explores the safety profile and potential clinical applications of lycopene. The present review underscores lycopene’s potential as a promising detoxification agent against AFB1 exposure, with the intent to stimulate further research and practical utilization in this domain.

## 1. Introduction

Mycotoxins are secondary toxic metabolites naturally produced by certain filamentous fungi. Approximately 500 mycotoxins have been identified, including aflatoxins (AFTs), deoxynivalenol (DON), T-2 toxin, HT-2 toxin, ochratoxin A (OTA), zearalenone (ZEN), nivalenol (NIV), and fumonisins (FBs) [1,2,3]. These mycotoxins can contaminate a variety of food items, encompassing fruits, grain crops, and processed products such as beer, dried fruits, cereals, and animal feed [1,4,5]. A recent report has indicated mycotoxins could be detected in Chinese herbal medicine [6]. Prior to 1985, the Food and Agriculture Organization (FAO) estimated that global food crop contamination from mycotoxins was around 25% annually. Currently, the figure may be up to 60–80%, based on detectable levels [2,7]. Developing countries face higher rates of mycotoxin contamination. For instance, Xu et al. found 100% positivity for DON, 68.7% for ZEA, and 99.5% for deoxynivalenol-3-glucoside (DON-3-G) among 370 wheat samples in Anhui Province, China [8]. Exposure to mycotoxins has been linked to various chronic diseases, including neurodegenerative issues, cardiovascular disease, chronic enteritis, and endemic diseases [9,10,11]. Acute mycotoxin poisoning can be fatal to both animals and humans [12]. Given the widespread contamination and significant health risks associated with mycotoxins, efforts to prevent, control, and detoxify these contaminants have emerged as a global imperative.

**Figure 1:**
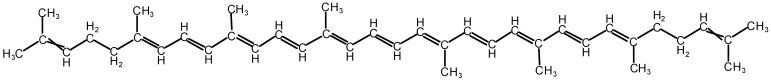
The chemical molecular structure of lycopene (i.e., C_40_H_56_).

Of particular concern among mycotoxins are AFTs, known for their potent toxicity and carcinogenic properties [13,14]. The primary producers of AFTs are *Aspergillus* (A.) *flavus* and *A. parasiticus* [15]. Over recent decades, a total of 21 AFTs have been identified, with the most prevalent variants being aflatoxin B1 (AFB1), B2 (AFB2), G1 (AFG1), G2 (AFB2), aflatoxin B1-8,9-epoxide (AFBO), M1 (AFM1), and M2 (AFM2) [1,16]. As early as 1987, the International Agency for Research on Cancer (IARC) had classified AFB1 and AFM1 as human Group 1 and Group 2B carcinogens, respectively [17]. Epidemiological data have shown a positive association between AFB1 exposure and liver cancer incidence, with 4.6–28.2% of liver cancer cases linked to AFB1 exposure [18,19,20]. Previous in vitro and animal studies have demonstrated that AFB1 exposure can lead to a range of toxic effects, including neurotoxicity, hepatotoxicity, cardiotoxicity, and gastrointestinal toxicity in mammals and poultry [21,22,23,24,25,26,27,28,29]. Mechanistic studies have revealed that AFB1-induced toxic effects involve various pathways, such as lipid peroxidation, inflammatory response, oxidative stress, and cell death (e.g., pyroptosis, apoptosis, necroptosis, and ferroptosis) mechanisms [1,30,31,32,33,34,35,36,37]. Further exploration by scientists reveals multiple signaling pathways, including the NOD-like receptor (NLR) family pyrin domain-containing 3 (NLRP3), aryl hydrocarbon receptor (AHR), phosphoinositide 3-kinase (PI3K)/protein kinase B (Akt), toll-like receptors (TLRs), adenosine 5′-monophosphate (AMP)-activated protein kinase (AMPK), the mammalian target of rapamycin (mTOR), tumor protein P53 (p53), mitogen-activated protein kinase (MAPK), peroxisome proliferator-activated receptor gamma (PPARγ) coactivator 1 alpha (PGC-1α), phosphatase and tensin homolog (PTEN)-induced kinase 1 (PINK1)/Parkin, Wnt/β-catenin, nuclear factor erythroid 2-related factor 2 (Nrf2)/kelch-like ECH-associated protein 1 (KEAP1), nicotinamide adenine dinucleotide phosphate (NADPH) oxidases (NOXs), nuclear factor-kappa B (NF-κB), and mitochondrial apoptotic pathways [1,30,31,32,33,34,35,36,37,38]. By targeting these critical signaling pathways, some compounds, including resveratrol, curcumin, caffeic acid, gallic acid, proanthocyanidin, and quercetin, have been demonstrated to own the potential detoxification effects against AFB1 exposure-induced toxicity by using in vitro cell or experimental animal models [1,39,40,41,42,43,44,45]. Several Chinese herbal extracts or probiotics could also effectively improve AFB1 exposure-induced toxic effects [42,46]. Recent studies have highlighted the protective effects of lycopene (Figure 1), a natural carotenoid, against AFB1 exposure-induced toxicity in animal models [47,48,49,50,51,52,53,54]. Lycopene, derived mainly from the diet, has shown promising safety profiles in both humans and animals and is commercially available in health care products in the U.S., China, and Europe [55,56,57]. This review aims to provide a comprehensive overview of lycopene’s protective effects against AFB1 toxicity, including its molecular mechanisms and clinical implications, to inform future research and interventions aimed at mitigating the health risks posed by AFB1 exposure.

## 2. An Overview of Lycopene’s Protections against Aflatoxin B1 Toxicity

Lycopene is a common non-provitamin, comprising a 40-carbon acyclic carotenoid with 13 double bonds and 11 linearly arranged conjugated double bonds. It is typically abundant in various vegetables and fruits, including watermelons, tomatoes, apricots, pink grapefruits, cranberries, papayas, guavas, and peaches [57,58]. Lycopene exhibits diverse biological activities such as anti-oxidative stress, anti-aging, anti-inflammatory, anti-cancer, and immune regulation functions [55,59,60,61,62]. The beneficial effects of lycopene in mitigating the harmful effects induced by AFB1 exposure are outlined in Table 1. Studies have demonstrated that the potential molecular mechanisms through which lycopene protects against AFB1-induced toxicity involve the inhibition of reactive oxygen species (ROS) production, the suppression of the inflammatory response, the mitigation of mitochondrial dysfunction, the enhancement of endogenous antioxidant levels and antioxidant enzyme activities, and the activation of the Nrf2/KEAP1 pathway and the PGC-1α pathway [47,48,49,50,51,52,53,54]. Furthermore, it was also reported that lycopene supplementation could influence the metabolism of AFTs by modulating cytochrome P450 (CYP450) isozymes in animals [63,64]. Subsequent sections will offer an in-depth examination of the precise molecular mechanisms responsible for the protective effects of lycopene.

### 2.1. Inhibition of Oxidative Stress

Oxidative stress generally occurs due to an imbalance between the body’s oxidative and antioxidant systems, often resulting in the overproduction of reactive oxygen species (ROS) [67]. ROS comprise various free radicals, such as superoxide anion (O_2_^•−^), hydroxyl radical (^•^OH), peroxynitrite (ONOO^−^), and nitric oxide (NO) [68]. Under normal physiological conditions, these free radicals are efficiently neutralized by intracellular endogenous antioxidants or antioxidant enzymes, including superoxide dismutase (SOD), glutathione peroxidase (GPX), catalase (CAT), and glutathione (GSH) [67]. Studies have shown that exposure to AFB1 can induce oxidative stress by promoting the generation of free radicals and reducing the levels of the antioxidants mentioned above or of antioxidant enzymes [40,69,70,71,72,73,74,75,76,77,78,79,80,81]. The generation of ROS by AFB1, like O_2_^•–^ and ^•^OH, is partially attributed to its metabolic processing in tissue [53]. Elevated levels of intracellular ROS can cause damage to lipids, proteins, DNA, and other cellular components, leading to various forms of cell death, such as apoptosis, autophagic cell death, ferroptosis, necroptosis, and necrosis, among others [82].

Lycopene has been reported to exhibit potent radical scavenging activities. Results from in vitro biochemical analyses have suggested that lycopene’s antioxidant properties are roughly twice as effective as curcumin, another powerful protective agent against AFB1 toxicity [1,83,84]. This potent radical scavenging activity is suggested to be associated with the number of conjugated double bonds in the structure of lycopene [85]. For example, a 2,2-Diphenyl-1-picrylhydrazyl (DPPH) and O_2_^•–^ radical scavenging test showed that the half maximal inhibitory concentrations (IC_50_s) of lycopene are approximately 20 and 5 µg/mL, respectively [86]. Lycopene supplementation has shown significant protective effects against AFB1-induced oxidative stress damage in various organs of mammals (e.g., mice and rats) and poultry (e.g., chicks and ducklings) [47,48,49,51,52,53,54,66]. For instance, El-Sheshtawy et al. demonstrated that a 25-day lycopene supplementation regimen markedly reduced AFB1-induced increases in intracellular malondialdehyde (MDA), a lipid peroxidation marker, while also enhancing the activities of glutathione S transferase (GST), catalase (CAT), and total antioxidant capacity (TAC), thereby attenuating AFB1-induced liver damage in ducklings [54]. Wan et al. observed that lycopene supplementation at 200 mg/kg via the basal diet significantly inhibited the production of reactive oxygen species (ROS) and lipid peroxidation products, such as MDA and 4-hydroxynonenal (4-HNE), in chicken liver tissue [48]. Huang et al. found that oral lycopene supplementation at 5 mg/kg per day for 30 days lowered MDA and H_2_O_2_ levels, while upregulating superoxide dismutase (SOD) and CAT activity, leading to a partial reduction in AFB1-induced testicular lesions in mice [47]. Furthermore, several studies indicated that lycopene supplementation effectively mitigated AFB1-induced oxidative stress in the livers, kidneys, and hearts of rodents by increasing GSH levels and the activities of GPX and thioredoxin reductase [22,48,51,53]. A recent study has shown that administering oral lycopene supplements at a dosage of 10 mg/kg body weight per day significantly reduced oxidative stress, mitochondrial dysfunction, and ferroptotic cell death caused by a combination of mycotoxins in the jejunum tissue of mice. These mycotoxins included ZEN at 10 mg/kg body weight, DOX at 1 mg/kg body weight, and AFB1 at 0.5 mg/kg body weight [87]. The study found that lycopene’s ability to activate the body’s antioxidant system played a crucial role in protecting against the toxic effects triggered by AFB1.

The Nrf2 belongs to the cap ‘n’ collar subfamily of basic region leucine zipper transcription factors and is recognized as a housekeeping gene that responds to oxidative stress triggered by xenobiotics [88]. In unstressed cells, Nrf2 interacts with KEAP1 in the cytoplasm. Under oxidative stress conditions, ROS and electrophiles can directly bind to KEAP1 at multiple sites, including cysteines 151 (C151), 273 (C273), and 288 (C288), facilitating the release of Nrf2 from KEAP1-mediated degradation and its translocation into the cell nucleus. Subsequently, Nrf2 activates the expression of over 200 cytoprotective genes involved in anti-inflammatory and antioxidant responses, phase II detoxification enzymes, and xenobiotic metabolism [89,90]. Previous studies have indicated that exposure to AFB1 can markedly suppress Nrf2 gene expression, leading to an increased sensitivity to AFB1 in Nrf2 knockout mice and underscoring the critical role of Nrf2 as a target of AFB1 [91]. Multiple studies have demonstrated that supplementation with lycopene effectively activates the Nrf2/KEAP1 pathway, leading to the upregulation of downstream genes such as SOD, CAT, glutathione S-transferase (GST), heme oxygenase-1 (HO-1), and quinone oxidoreductase 1 (NQO1), among others. This activation provides protection against various drugs (including colistin, cisplatin, and atrazine), environmental toxins (such as aristolochic acid, atrazine, and chlorpyrifos, Di[2-ethylhexyl]phthalate), and tissue damage induced by ischemia-reperfusion [92,93,94,95,96,97]. Huang et al. recently reported that oral supplementation with 5 mg/kg of lycopene per day for 30 days significantly increased the expression of nuclear Nrf2 protein in the testicular tissues of mice [47]. Yu et al. found that lycopene supplementation at the same dosage for the same duration notably upregulated Nrf2 and its downstream targets, including CAT, NQO1, SOD1, glutathione synthetase (GSS), glutamate–cysteine ligase catalytic (GCLC), and glutamate–cysteine ligase modifier subunit (GCLM), thereby mitigating AFB1-induced renal damage in mice [22]. In a chick model, researchers demonstrated that lycopene supplementation at a basal diet level (200 mg/kg) significantly upregulated Nrf2 expression and downstream genes like HO-1, Cu/ZnSOD, MnSOD, CAT, and GPX mRNAs, leading to a pronounced reduction in AFB1-induced intestinal damage [52]. These results suggest that the activation of the Nrf2/KEAP1 pathway plays a role in the protective effects of lycopene against oxidative stress induced by AFB1 exposure. Previous studies have suggested that lycopene-induced activation of Nrf2/KEAP1 may be modulated by p62, AMP-activated protein kinase (AMPK), and silent information regulator 1 (SIRT1) [95,98,99]. Nonetheless, the direct interaction of lycopene with KEAP1 remains unclear, highlighting the need for further investigations to elucidate the precise molecular mechanisms of lycopene in activating the Nrf2/KEAP1 pathway.

**Figure 2:**
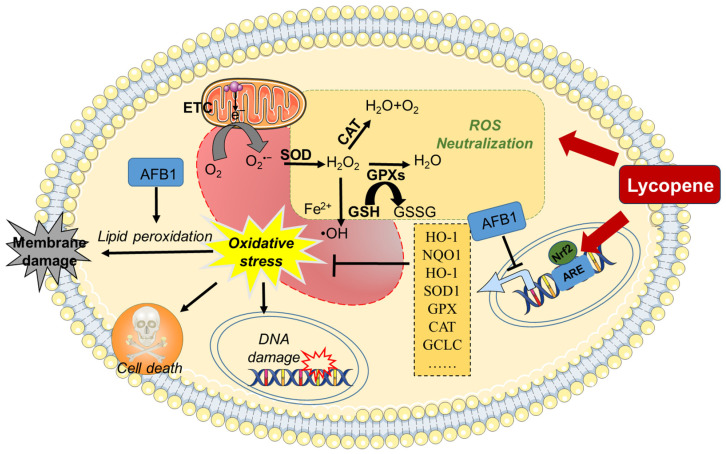
A working model of lycopene protecting against AFB1-induced oxidative stress. AFB1 exposure could induce oxidative stress damage through the inhibition of antioxidant enzymes’ activities, via the inhibition of the Nrf2 pathway. Lycopene supplementation could offer a protection for AFB1 exposure-induced production of ROS and oxidative stress damage by activating the Nrf2 pathway and enhancing intracellular antioxidant enzyme activity and antioxidant levels. AFB1, aflatoxin B1; ARE, antioxidant response element; CAT, catalase; ETC, electron transport chain; GCLC, glutamate–cysteine ligase catalytic subunit; GSSG, oxidized glutathione; GPX, glutathione peroxidase; GSH, glutathione; H_2_O_2_, hydrogen peroxide; HO-1, heme oxygenase 1; NQO1, NAD (P)H quinone oxidoreductase 1; Nrf2, nuclear factor erythroid 2-related factor 2; O_2_^•–^, superoxide anion; ^•^OH, hydroxyl radical; ROS, reactive oxygen species; SOD, superoxide dismutase.

In summary, supplementation with lycopene may provide robust protection against AFB1-induced oxidative stress damage by eliminating ROS, enhancing antioxidant enzyme activity, and activating the Nrf2/Keap1 pathway (see Figure 2).

### 2.2. Improvement in Inflammatory Response and Immune Function

Prior investigations have demonstrated that exposure to AFB1 can activate an inflammatory response [72,100,101,102]. Epidemiological studies have established a positive association between AFB1 exposure and various chronic diseases, such as neurodegenerative disease, chronic enteritis, and liver cancer [16,100,103,104]. Experimental evidence suggests that AFB1 exposure can enhance the development of lung tumorigenesis and liver cancer by activating inflammatory responses, particularly pronounced in individuals co-infected with hepatitis B and C viruses [38,105,106,107,108,109,110]. Furthermore, chronic exposure to AFB1 can lead to immunosuppression, as evidenced by decreased spleen weights, lymphocyte reduction (B and T cells), and decreases in immune cells and immune-related factors like IL-2, IL-10, and interferon-gamma (IFN-γ) [26,111,112]. The potential molecular mechanisms underlying AFB1-induced inflammatory responses and immunotoxicity may involve various pathways, such as the protein kinase C (PKC), AHR, NF-κB, toll-like receptor 4 (TLR4), NRLP3, MAPK, and receptor-interacting serine/threonine-specific protein kinase 1 (RIPK1) pathways [1,38,113,114,115].

Various studies have demonstrated the immune-regulatory and anti-inflammatory properties of lycopene. Its effectiveness is attributed to its lipophilic nature, which allows it to modulate signaling pathways of inflammatory mediators and induce the expression of antioxidant genes by interacting with cellular components [116]. Xu et al. demonstrated that oral lycopene supplementation at a dose of 5 mg/kg per day for 30 days significantly reduced AFB1-induced histopathological injuries in mouse spleens; meanwhile, lycopene supplementation notably increased spleen weight, spleen coefficient, T lymphocyte subsets, and upregulated the mRNA expressions of TNF-α, IFN-γ, and IL-2 genes in the spleen and the corresponding proteins in the bloodstream [26]. Another study by Sarker et al. showed that lycopene supplementation through diet (200 mg/kg feed) for 42 days effectively inhibited increases in IFN-γ, IL-1β proteins, and mRNA expression, caused by AFB1 exposure, upregulated levels of IL-10 protein in the intestine mucosa of chicks, enhanced intestinal barrier function, and improved intestinal health [52]. Lycopene is also an inhibitor of NF-κB, which is a critical transcription factor mediating the expression of multiple inflammatory factors, including TNF-α, IL-1β, COX2, and IL-6 [117]. The inhibitory effects of lycopene on the NF-κB pathway could be mediated via the blockade of the MAPK and TLR4 pathways [118,119]. In addition, excessive ROS production could exacerbate the generation of pro-inflammatory cytokines and chemokines via triggering the PKC, NLRP3, TLR4, NF-κB, and MAPK pathways [16,120].

**Figure 3:**
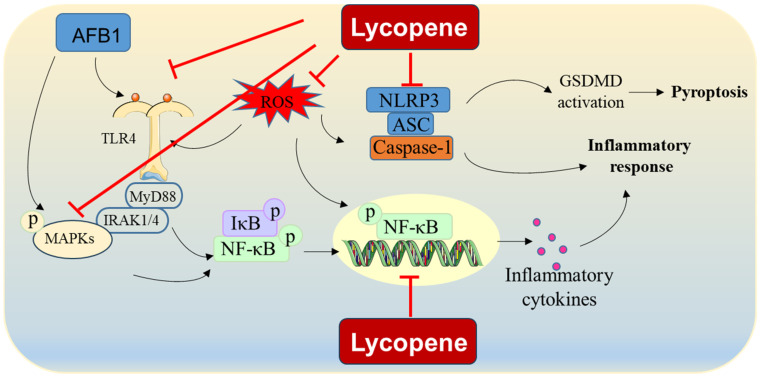
AFB1-induced inflammatory response and proposed modulation of lycopene. Lycopene supplementation could reduce AFB1 exposure-induced inflammatory response via the inhibition of formation of NLRP3-mediated inflammasome, NF-κB pathway, MAPK pathway, and NLRP3 pathway. It also may be partly attributed to the inhibition of ROS production.

Taken together, these results indicate that lycopene may mitigate AFB1-induced inflammatory responses by inhibiting the TLR4, NF-κB, and MAPK pathways and reducing ROS production (Figure 3). However, the precise molecular mechanisms responsible for lycopene’s effectiveness in countering AFB1-induced inflammation remain incompletely understood, highlighting the need for further comprehensive investigations into these mechanisms.

### 2.3. Inhibition of Mitochondrial Dysfunction and Apoptosis

Mitochondria play a crucial role in sustaining life by facilitating the production of adenosine triphosphate (ATP) through oxidative phosphorylation (OXPHOS) [121]. They are also involved in regulating apoptotic cell death processes [121]. Dysfunctions in mitochondria, resulting from sustained damage, can affect various cellular processes such as mitochondrial membrane potential, respiration, and electron transfer [122,123]. This dysfunction can be triggered by factors like drugs, toxins, and excessive production of reactive oxygen species (ROS), ultimately leading to cell apoptosis and other forms of regulated cell death [121]. Studies have demonstrated that exposure to aflatoxin B1 (AFB1) can induce mitochondrial dysfunction, as evidenced by disruptions in mitochondrial DNA, electron transport chain, membrane potential, and biosynthesis [48,124,125,126]. Notably, even low doses of AFB1 exposure can lead to abnormal changes in mitochondrial structure, membrane potential, and expression of genes involved in electron transport chain complexes in the liver of mice [127]. Moreover, AFB1 exposure has been linked to the diminished mRNA expression of key regulators, such as nuclear respiratory factor 1 (Nrf1), mitochondrial transcription factor A (MTFA), PGC-1α, and peroxisome proliferator-activated receptor γ coactivator 1α (PPAR1α), important for mitochondrial biosynthesis [48,127,128]. Notably, MTFA is a direct regulator of mitochondrial DNA replication/transcription. The activation of PGC-1α could stimulate a powerful expression of Nrf1 and Nrf2 genes; it also binds to Nrf1 and coactivates the expression of MTFA, promoting mitochondrial DNA replication/transcription [129]. These data indicate that AFB1 exposure-induced abnormalities of mitochondrial biosynthesis may involve the inhibition of the PGC-1α/Nrf1/MTFA pathway.

Recently, Huang et al. found that oral lycopene supplementation at the dose of 5 mg/kg per day for 30 days could effectively improve AFB1 exposure to the damage in mitochondrial structure and upregulate the expression of PGC-1α, Nrf1, MTFA, and cytochrome C oxidase IV (COXIV) mRNAs in the testicular tissues of mice [47]. Similarly, Wan et al. found that lycopene supplementation at 200 mg/kg via the basal diet for 42 days significantly improved mitochondrial function, evidenced by reduced swelling, increased activities of mitochondrial complexes III, IV, and V, elevated ATP levels, and enhanced expression of PGC-1α, Nrf1, and MTFA mRNAs in AFB1-treated chickens’ livers [48]. A previous study found that lycopene-mediated upregulation of PGC-1α is partly dependent on the expression of sirtuin 1 (SIRT1), which could deacetylate PGC-1α at multiple lysine sites and consequently increase the activities of PGC-1α [130]. In addition, the activation of AMPK by lycopene could enhance PGC-1α-dependent transcription via the phosphorylation of SIRT1 [131]. These evidences indicated that the activation of PGC-1α pathways may be dependent on the activation of AMPK and SIRT1 proteins. Furthermore, Xu et al. demonstrated that 5 mg/kg per day of oral lycopene supplementation for 30 days mitigated AFB1-induced loss of mitochondrial membrane potential in mouse spleen tissues [26]. In conclusion, the evidence suggests that lycopene supplementation can ameliorate AFB1-induced mitochondrial damage, loss of membrane potential, and dysfunction of the ETC and biosynthesis processes. The precise molecular mechanisms still need to be further investigated.

Generally, an increase in the ratio of the pro-apoptotic protein Bax to the anti-apoptotic proteins Bcl-2 or Bcl-XL can lead to the formation of mitochondrial permeability transition pores (MPTPs), the subsequent release of CytC from the mitochondria, the activation of caspase-9 and caspase-3, and ultimately culminate in cell apoptosis [132]. Previous research has shown that exposure to AFB1 significantly elevates the Bax/Bcl-2 protein ratio, enhances caspase-9 and caspase-3 activities, and increases their mRNA expressions, ultimately resulting in cell apoptosis in HepG2 cells, IMR-32 cells (a neuroblastoma cell line), as well as in the brain, spleen, liver, and renal tissues of animals [26,80,133,134,135]. Numerous studies have indicated that lycopene supplementation can effectively mitigate apoptotic cell death induced by colistin, tert-butyl hydroperoxide, and lead through the inhibition of mitochondrial dysfunction and the mitochondrial pathway [94,136,137]. Consistent with these findings, researchers have observed that lycopene supplementation at different doses can successfully attenuate AFB1 exposure-induced cell apoptosis, as evidenced by the suppression of cytoplasmic CytC, Bax, and cleaved caspase-3 proteins’ expression, as well as the activities and mRNA expression of caspases-3 and -9, and the upregulation of Bcl-2 protein [26,47,87,138].

**Figure 4:**
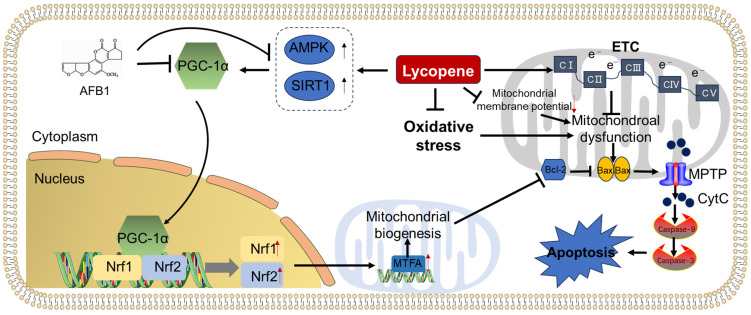
Lycopene supplementation attenuated AFB1-induced mitochondrial dysfunction and apoptotic cell death. AFB1 exposure could inhibit the activation of PGC-1α directly or indirectly via the inhibition of AMPK and SIRT1 proteins, then reduce the mitochondrial biogenesis via the Nrf1/Nrf2/MTFA pathway, and, finally, ameliorate mitochondrial dysfunction and apoptosis. Lycopene supplementation could protect AFB1 exposure-induced mitochondrial dysfunction and apoptosis via the activation of the PGC-1α pathway, the inhibition of oxidative stress and the loss of mitochondrial membrane potential, and the activation of the mitochondrial apoptotic pathway.

A proposed model illustrating the protective effect of lycopene supplementation against AFB1 exposure-induced mitochondrial dysfunction and mitochondrial apoptotic pathway is presented in Figure 4. Notably, the ameliorated effects of lycopene supplementation on AFB1 exposure-induced mitochondrial dysfunction may be partly attributed to the activation of PGC-1α/Nrf1/MTFA pathway-mediated mitochondrial biosynthesis, the improvement in ETC function, and the increases in mitochondrial membrane potential. Additionally, oxidative stress is a critical contributor for mitochondrial dysfunction [139]. Therefore, the inhibitory effects of lycopene on the production of ROS may also play a critical role. Nevertheless, the specific molecular mechanisms underlying lycopene’s efficacy against AFB1-induced mitochondrial dysfunction remain largely unclear, the detailed exploration for these mechanisms are still required.

### 2.4. Metabolic Intervention

The liver plays a dual role in the metabolism of aflatoxin B1 (AFB1), serving as both a target for its toxic effects and a crucial organ for its detoxification in humans and animals [1,140]. Variations in the metabolism of AFB1 among species and organs are attributed to differences in the expression and content of metabolic enzymes [141]. Dohnal et al. provide a comprehensive review of these differences. AFB1 undergoes four major pathways of metabolism, including hydroxylation, ketoreduction, O-dealkylation, and epoxidation [142]. Broadly, four major pathways have been identified in the metabolism of AFB1, including hydroxylation, ketoreduction, O-dealkylation, and epoxidation [141]. Approximately 95% of AFB1 undergoes transformation into highly toxic AFBO and AFM1, as well as other less toxic forms (e.g., AFP1, AFK1, or AFB2a), by cytochrome P450 (CYP450) enzymes (such as CYP1A1, CYP1A2, CYP1A5, CYP2A6, CYP2A13, CYP3A37, and CYP3A4) in the liver tissues [141,143,144,145,146]. Notably, AFBO is considered the primary toxic metabolite of AFB1 due to its direct interaction with DNA, leading to multiple toxic effects within cells [147]. AFBO can be detoxified through conjugation with glutathione (GSH) or through hydrolysis by epoxide hydrolase enzymes, to produce the highly cytotoxic AFB1–8,9-dihydro diol (AFB1–dhd) [141].

**Figure 5:**
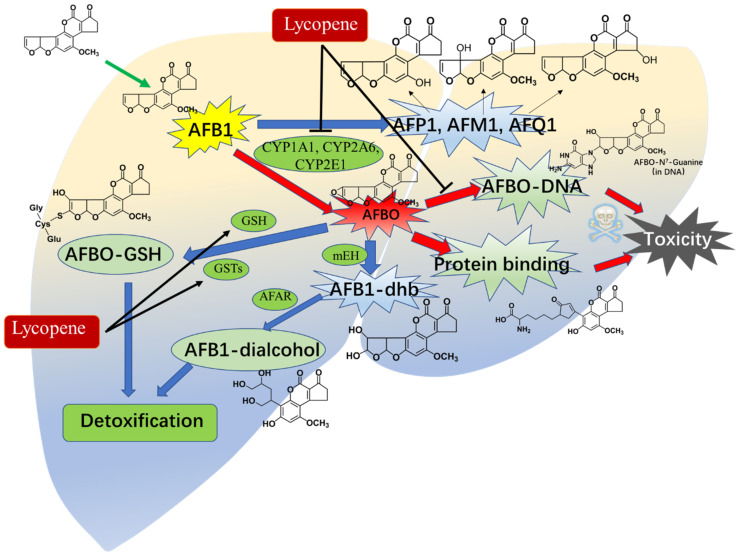
An overview of lycopene supplementation modulating the metabolism of AFB1 in animals’ livers. Lycopene supplementation could reduce the formation of AFBO, AFP1, AFM1, AFQ1, and AFB1–DNA adducts in the body via the inhibition of CYP1A1, CYP2A6, and CYP2E1 enzyme activities in the liver tissues of animals. It also could upregulate the level of GSH and phase II enzyme GSTs, enhance the formation of AFBO–GSH and AFB1–dialcohol, and, finally, promote the detoxification of AFB1.

Previous studies have indicated that lycopene can modulate the activities of metabolic enzymes, including CYP3As, CYP2C, CYP2D, and CYP2E [148]. Nosková et al. reported that oral supplementation of lycopene at doses ranging from 4 to 100 mg/kg per day for 10 days could enhance the activities of CYP2B, CYP2D, and CYP3A [149]. Wan et al. demonstrated that lycopene supplementation at a dose of 200 mg/kg in the basal diet for 42 days significantly decreased the activities of CYP1A1 and CYP2A6, albeit not affecting CYP1A2 and CYP3A4, thereby leading to a reduction in the formation of AFB1–DNA adducts and DNA damage in the liver tissues of chickens [64]. Lin et al. observed that pretreatment with lycopene at a dose of 10 mg/kg per day through oral supplementation for 14 days markedly decreased the expression of hepatic CYP2E1 protein, consequently lowering AFB1-induced liver toxicity [150]. Moreover, Tang et al. found that oral administration of lycopene at a dose of 100 mg/kg/day for 15 days significantly reduced the formation of AFB–DNA adducts in liver tissues, as well as the levels of AFM1, AFQ1, and AFP1 excreted in urine, along with AFB1–albumin adducts in serums of rats [49]. Additionally, AFB1-induced AFB1–N7-guanine adducts could lead to apoptotic cell death and the suppression of the p53 protein expression [138]. Reddy et al. reported that lycopene supplementation at a dose of 0.5 μg/mL notably diminished the formation of AFB1–N7-guanine and its excretion in HepG2 cells [138]. Correspondingly, lycopene supplementation also markedly decreased the levels of AFB1–N7-guanine in rat urine and elevated levels of AFB–NAC in urine excretion [49]. AFB–NAC is the primary detoxifying metabolic product of AFBO, and its formation is reliant on the activities of phase II enzymes, including GPX and GST [151]. This observation aligns with prior studies indicating that lycopene can elevate GSH levels and the activity of enzymes such as GPX, GST, and glutathione reductase (GR) in tissues exposed to AFB1 (e.g., kidney, heart, liver, and intestine) across various animal models including chickens, ducklings, rats, and mice [22,51,52,53,54]. These findings suggest that lycopene may protect against AFB1 toxicity by modulating the activities of CYP450 enzymes and phase II detoxification enzymes (see Figure 5).

## 3. Safety of Lycopene and Its Clinical Application

Lycopene, a potent antioxidant found in tomatoes and tomato-based products, is a significant component of human dietary intake, typically ranging from 0.7 to 25.2 mg/day [152,153]. The absorption of lycopene from the diet typically ranges from 10% to 30% of the intake amount, with the remainder excreted [153]. Studies have indicated that, in humans, the time to reach maximum concentration (tmax) and the elimination half-life (t1/2) of lycopene are 0.5 and 48 days, respectively. Numerous clinical trials have demonstrated that lycopene supplementation offers various health benefits in the prevention and treatment of chronic diseases, such as cancer, high-density lipoprotein (HDL)-associated inflammation, and heart disease [154,155,156]. Experimental studies have shown that oral supplementation of lycopene, at doses ranging from 5 to 200 mg/kg/day, can have multiple biological effects, such as antioxidant, anti-aging, anti-inflammatory, immune regulation, and antimicrobial functions [157,158,159,160]. Previous studies have shown that lycopene has a high safety profile. An older study reported that a subcutaneous injection of lycopene at a dose of 3 g/kg body weight led to only a temporary decrease in muscle tone in mice, while oral and intraperitoneal administration of this dose had no effect [161]. Moreover, recent research by Michael and colleagues demonstrated that rats treated with lycopene beadlet formulations, at doses up to 500 mg/kg body weight/day for 14 weeks or 1000 mg/kg body weight/day for 4 weeks, showed no significant toxicity, establishing a non-observable adverse effect level (NOAEL) of 500 mg/kg body weight/day for lycopene [162]. These findings suggest that the effective dose of lycopene is below its safety threshold, making it a viable option in both medical and food industries [154,155,156,163].

While animal studies have shown the effectiveness of lycopene in mitigating AFB1 exposure-induced toxicity, limited clinical evidence exists regarding its detoxification effects against AFB1 in humans. Therefore, further clinical trials or subclinical investigations are necessary to fully understand the detoxification potential of lycopene against AFB1.

## 4. Conclusions and Future Directions

The global concern regarding AFB1 contamination arises from its significant toxicity and carcinogenicity in both human and animal populations. Extensive studies, encompassing both animal experimentation and epidemiological inquiries, have elucidated the various adverse effects associated with AFB1 exposure. These effects include, but are not limited to, neurotoxicity, immune toxicity, reproductive toxicity, genotoxicity, hepatotoxicity, nephrotoxicity, and gastrointestinal toxicity in mammals and poultry. Notably, lycopene, a lipid-soluble natural carotenoid obtained from the daily diet, has shown promise in protecting against the multiple toxic effects induced by AFB1 exposure, such as immune toxicity, reproductive toxicity, genotoxicity, hepatotoxicity, nephrotoxicity, cardiac toxicity, and hematologic toxicity. The underlying molecular mechanisms of this protection involve the inhibition of oxidative stress, inflammation, lipid peroxidation, CYP450 enzyme activity, and the mitochondrial apoptotic pathway, as well as the activation of mitochondrial biogenesis, the endogenous antioxidant system, and the Nrf2/KEAP1 and PGC-1 pathways. Moreover, supplementation with lycopene has demonstrated the ability to enhance gut health and immune function.

These findings collectively suggest that lycopene holds promise as a potent therapeutic agent against AFB1-induced toxic effects in mammals and poultry. However, despite these promising results, the precise protective mechanisms of lycopene remain incompletely understood, and there is a dearth of effective clinical evaluations regarding its protective effects against AFB1-induced harm in both human and animal populations. Furthermore, addressing the issue of lycopene’s bioavailability is crucial. Consequently, additional research efforts are warranted to gain a comprehensive understanding of the molecular basis of lycopene’s protective capabilities, its bioavailability, and its clinical efficacy.

## Figures and Tables

**Table 1:** A summary of lycopene protecting against AFB1 exposure-induced harmful effects in vivo and in vitro.

Animal Models	Treatments	Protective Effects of Lycopene	Refs.
Male Kunming mice	Mice were orally administrated with AFB1 at the dose of 0.75 mg/kg/day or cotreated with lycopene at the dose of 5 mg/kg/day. All mice were treated for consecutive 30 days.	Lycopene supplementation significantly attenuated AFB1 exposure-caused lesions of testicular microstructure and ultrastructure, and sperm abnormalities in mice. Meanwhile, lycopene supplementation significantly ameliorated AFB1 exposure-induced oxidative stress and the functional deficiency of mitochondrial biosynthesis, and significantly activated the Nrf2 pathway and the PGC-1α pathway in the testicular tissue of mice.	[47]
One-day-old Pekin ducklings	The ducklings were fed a ration contaminated with 30 ppb (equal to 30 μg/kg body weight) of AFTs (a mixture containing AFB1 and other AFTs) for 2 weeks and co-treated with or without lycopene, at the final dose of 100 mg/kg body weight. After AFT treatment, the ducklings were orally fed continually for an additional 10 days.	Lycopene supplementation markedly attenuated AFTs exposure-induced liver dysfunction. Lycopene supplementation also significantly increased the levels of total antioxidant capacity (TAC), catalase (CAT), and glutathione S-transferase (GST) activities, and significantly decreased the levels of malondialdehyde (MDA), finally effectively improving AFTs exposure-induced hepatic oxidative stress damage. Meanwhile, lycopene treatment significantly decreased the residues of AFTs in the liver tissue.	[54]
Male F344 rats	Rats were orally administrated with AFB1 at the final dose of 250 μg/kg body weight daily and co-treated with lycopene at the final dose of 100 mg/kg body weight daily. All rats were treated for 3 weeks (5 days per week).	Lycopene treatment markedly attenuated AFB1 exposure-induced toxic symptoms, including weakness, anorexia, bloody urine, ascites, and ataxia. In addition, gross necropsy and histopathological examination found lycopene treatment marked decreased AFB1 exposure-caused necrosis, hepatotropism, fatty infiltration, and bile duct epithelium hyperplasia in liver tissue. In addition, lycopene treatment greatly modulated AFB1 metabolism and metabolic activation, and significantly reduced formation of AFB1–DNA adducts.	[49]
One-day-old male Arbor Acres broiler chicks	Chicks were orally fed with a 100 µg/kg AFB1-contaminated basal diet and co-fed with or without lycopene (purity ≥ 80%) with a 200 mg/kg basal diet. All chicks were treated for 42 days.	Lycopene supplementation significantly improved the liver function of AFB1-treated chicks. It significantly decreased the levels of H_2_O_2_ and reactive oxygen species (ROS) levels, and significantly increased the levels of GSH and the activities of superoxide dismutase (SOD), thioredoxin peroxidase (TPX), and glutathione peroxidase (GPX) in AFB1-treated liver tissue. Meanwhile, lycopene supplementation significantly attenuated AFB1 exposure-induced mitochondrial dysfunction and the functional loss of mitochondrial biogenesis, increased the activities of mitochondrial electron transfer chain complexes, and activated the PGC-1α pathway. Lycopene supplementation decreased the intestinal villus height (VH) and crypt depth ratio (VCR) while increasing the crypt depth. Lycopene supplementation could also decrease the activities of cytochrome P450 (CYP450) isozymes (e.g., CYP1A1 and CYP2A6), then reduced the formation of AFB1–DNA in the liver tissue of chicks.	[48,64]
One-day-old male Arbor Acres broilers	Chicks were orally fed with a 100 µg/kg AFB1-contaminated basal diet and co-fed with or without lycopene (purity ≥ 80%) with a 200 mg/kg basal diet. All chicks were treated for 42 days.	Lycopene treatment significantly increased the levels of interleukin (IL)-10 protein and downregulated the expression of IL-1β mRNA, as well as attenuating the inflammatory response in the jejunum tissue of AFB1-treated chicks. Moreover, lycopene supplementation also significantly attenuated AFB1 exposure-induced oxidative damage in the jejunum tissue of chicks.	[52,65]
Male Wistar-Albino rats	Rats were orally administrated with AFB1 at a dose of 0.5 mg/kg/day for 7 days and lycopene at a dose of 5 mg/kg/day, for 15 days.	Lycopene supplementation markedly attenuated AFB1 exposure-induced liver dysfunction and liver oxidative damage through upregulating the levels of antioxidants and the activities of antioxidant enzymes.	[51]
Male Wistar-Albino rats	Rats were orally administrated with AFB1 at the dose of 0.5 mg/kg/day for 7 days or 1.5 mg/kg/day for 3 days, and all AFB1-treated rats were treated with or without lycopene at a dose of 5 mg/kg/day for 15 days.	Lycopene supplementation significantly attenuated AFB1 exposure-induced pathological changes in the kidney and heart tissues of rats. It also significantly inhibited AFB1 exposure-induced lipid peroxidation and upregulated antioxidant enzyme activities in the kidney and heart tissues of rats.	[53]
Male Kunming mice	Mice were orally administrated with AFB1 at the dose of 0.75 mg/kg body weight per day and co-treated orally with lycopene at the dose of 5 mg/kg body weight per day. All mice were treated for 30 days.	Lycopene supplementation significantly protected against AFB1-induced erythrocyte dysfunction and spleen toxicity via the inhibition of the inflammatory response, oxidative stress, and the mitochondrial apoptotic pathway and via the improvement in immune function in mice.	[26,66]
